# Multidimensional cognitive functioning across sport classifications: insights from high-performance athletes

**DOI:** 10.3389/fpsyg.2026.1837191

**Published:** 2026-09-23

**Authors:** Carmel Camilleri, Kathryn Johnston, Nick Wattie, Joseph Baker, Magdalena Wojtowicz

**Affiliations:** 1 Department of Psychology, York University, Toronto, ON, Canada; 2 Tanenbaum Institute for Science in Sport, Faculty of Kinesiology and Physical Education, University of Toronto, Toronto, ON, Canada; 3 Faculty of Health Sciences, Ontario Tech University, Oshawa, ON, Canada

**Keywords:** computerized neuropsychological test, high-performance athletes, multidimensional cognitive profiles, multivariate base-rates, sport classification

## Abstract

Recent research indicates that high-performance athletes may exhibit superior cognitive functioning, yet much of this work has examined single domains in isolation, without considering how cognitive profiles may differ across types of sport. To address this gap, the current study adopts a multi-domain framework to examine domain-general cognitive functioning in high-performance athletes. The sample consisted of 188 athletes from a regional level training/development organization (n_Female_ = 109, n_Male_ = 79; Mean age ± SD: 16.56 ± 1.95 years). Athletes completed a battery of computerized neuropsychological tests assessing a broad range of cognitive functioning, including aspects of working memory, inhibition, attention/concentration, and spatial processing. Athletes from team sports outperformed those in speed/strength and precision/skill-dependent sports, particularly in tasks assessing visual short-term (working) memory (*p* < 0.001), response inhibition (*p* < 0.001), visuospatial working memory (*p* < 0.001), and working memory (*p* < 0.001). When looking at the proportion of high scores achieved across sport classifications, most athletes achieved at least one high score (75th: 83.9–94.7%), though proportions declined at higher cutoffs (91st: 42.9–60.5%). Chi-square analyses revealed that team sport athletes consistently outperformed the other groups in achieving multiple high scores, particularly at the 75th (*p* < 0.002) and 84th (*p* < 0.001) percentile cutoffs. These findings provide a foundation for future research to advance our understanding of how sport-specific demands may be associated with cognitive functioning.

## Introduction

Previous research has established a positive relationship between exercise, overall physical activity, and cognition (Chan et al., 2011; Diamond and Ling, 2016; Etnier and Chang, 2009; Frederiksen et al., 2014; Krenn et al., 2018; Marchetti et al., 2015). Notably, studies have demonstrated both high-performance athletes and individuals with greater sport expertise exhibit superior domain-general cognitive functioning than their lower-sport expertise counterparts (Beavan et al., 2020; Cona et al., 2015; Faubert, 2013; Romeas et al., 2016; Verburgh et al., 2014; Vestberg et al., 2012). Domain-general cognitive functioning involves a range of cognitive processes, including executive functions (EF), attention, or processing speed, all of which are vital for adapting, managing, and integrating information across different tasks and situations (Vona et al., 2024). These cognitive processes likely enable athletes to efficiently process and adapt to incoming information, enhancing their capacity for rapid decision-making and high performance in pressure-filled situations. As performing in sports requires a wide range of cognitive processes, prior work has primarily focused on assessing athletes’ EF [e.g., (Alves et al., 2013; Faubert, 2013; Fleddermann et al., 2023)]. EF encompasses a set of cognitive processes that allow individuals to plan, organize, and regulate behavior, while adapting to changing situations. These processes are also underpinned by distinct neural substrates, with neuroimaging and electrophysiological research linking EF performance to prefrontal cortical activation and dissociable large-scale brain networks (Hampshire et al., 2012; Takahashi and Grove, 2023), while also demonstrating that EF-related neural activity (e.g., event-related potentials indexing inhibitory and proactive control) differs with sport expertise and can be modulated through direct cortical stimulation (Bianco et al., 2017; Di Russo et al., 2010; Kamali et al., 2023). This broad cognitive domain includes components necessary for fluid intelligence processes, including the ability to reason, solve problems, and plan goal-directed behavior (Diamond and Ling, 2016; Karr et al., 2018). While these skills are essential for regulating behavior, adapting to changing situations, and planning goal-directed actions (Diamond and Ling, 2016), other cognitive domains, such as visuospatial skills, attention, and processing speed, have received comparatively less attention (Cona et al., 2015; Rahimi et al., 2022; Vestberg et al., 2012). These abilities may be especially important in sports demanding rapid visual tracking, spatial awareness, and quick decision-making. While sport expertise has been linked to enhanced cognitive performance, few studies have explored how these advantages translate into on-field performance (Elferink-Gemser et al., 2018; Vestberg et al., 2017). Uncovering the cognitive mechanisms behind high-performance could inform training strategies that integrate cognitive development to enhance athletic success.

Several theoretical frameworks have been proposed to explain the relationship between cognition and performance in athletes. While our study does not directly test these frameworks, they provide a conceptual backdrop for understanding how cognitive functioning may relate to athletic performance, including the cognitive skill transfer, narrow transfer, and broad transfer hypotheses. The cognitive skill transfer hypothesis posits that individuals who train on a particular cognitive task may also enhance their performance on related, but untrained cognitive tasks (Jacobson and Matthaeus, 2014; Voss et al., 2010). According to this hypothesis, engaging in cognitively demanding activities, such as those required in high-level sports, strengthens underlying cognitive processes, which then transfer to performance in other, non-practiced domains. Moreso, the narrow transfer hypothesis suggests that expertise in a specific activity, such as a particular sport, enhances cognitive skills directly related to sport specific demands (e.g., visuospatial processing in hockey or reaction time in tennis) while providing little to no benefit for unrelated cognitive tasks (Furley and Memmert, 2011). For instance, athletes in sports like hockey or tennis may have enhanced visuospatial processing, or attentional abilities, but these processes are closely tied to the specific demands of their sport. As such, this theory posits that these skills rarely transfer to unrelated tasks, since the cognitive skills are finely tuned to sport-specific cues rather than broadly applicable abilities. Similarly, the broad transfer hypothesis suggests prolonged training in a particular activity (e.g., hockey, diving) improves cognitive functioning beyond domain-specific tasks, leading to measurable advantages across a range of cognitive functions (Allen et al., 2011; Voss et al., 2010). This theory is further supported by four meta-analyses demonstrating that higher levels of sport expertise are linked to superior cognitive functioning on tasks assessing domain-general cognitive functioning (Kalén et al., 2021; Logan et al., 2022; Scharfen and Memmert, 2019; Voss et al., 2010). Past research has reported superior cognitive functioning in athletes (i.e., primarily young adults (ages 18–30) in open-skill team sports such as soccer, basketball, and hockey) on various ‘domain-general’ cognitive tasks, including but not limited to EF (i.e., working memory, cognitive flexibility, and inhibition), attention, visuospatial processing, and processing speed (Alves et al., 2013; Elferink-Gemser et al., 2018; Faubert, 2013; Vaughan and Laborde, 2020; Verburgh et al., 2014). These frameworks are presented here as theoretical context for interpreting group differences in cognitive performance across sport classifications; as the present study is cross-sectional, it is not designed to adjudicate between these transfer mechanisms.

Previous research on the relationship between cognitive functioning and sport expertise has primarily focused on athlete and non-athlete populations, demonstrating that athletes outperform non-athletes in various cognitive aspects, with differing levels of sports experience playing a key role in enhancing cognitive functioning (Burris et al., 2019; Diamond and Ling, 2016; Jacobson and Matthaeus, 2014). For instance, research examining EF in youth soccer players revealed non-significant effects across different levels of sport expertise (Beavan et al., 2020). These discrepancies may arise from factors such as small sample sizes, variations in task validity, evolving cognitive assessments, and the size of reported effects (Furley and Memmert, 2011; Vona et al., 2024). Building on this, researchers have also explored how the specific type of sport athletes engage in influences their cognitive functioning, offering deeper insights into how varying sport demands may be associated with cognitive profiles.

A widely used classification system is open skill and closed skill sports. Open skill sports require athletes to react in dynamic, changing, and unpredictable environments that are externally paced (e.g., hockey, soccer). In contrast, closed skill sports require athletes to engage in a highly consistent, predictable and self-paced sporting environments (Allard and Burnett, 1985) (e.g., diving, swimming). When studies use the open and closed-skill classification framework, individuals from open-skill sports consistently perform better on a variety of cognitive tasks than closed-skill athletes (Holfelder et al., 2020; Russo et al., 2022; Takahashi and Grove, 2023; Zhu et al., 2022). A more refined classification system was created that included three separate sport classifications: strategic, interceptive, and static (Voss et al., 2010). Strategic sports require athletes to adapt to highly varying situations while considering teammates, opponents and other objects in an externally paced environment (e.g., hockey). Interceptive sports require athletes’ dynamic coordination between their body and their environment (e.g., tennis), while static sports are self-paced activities in highly consistent circumstances (e.g., swimming). When using this classification of sport, associations between cognition and sport have been more inconclusive. For instance, some studies have demonstrated that strategic sport athletes have superior performance in tasks relating to processing speed (Voss et al., 2010), while others have demonstrated enhanced performance on tests assessing aspects of EF and attention (Krenn et al., 2018; Meng et al., 2019; Vestberg et al., 2012). The current study employs a more fine-tuned sport classification framework that incorporates athletes physiological, tactical, skill, and cognitive demands in their classification of sport. This classification was chosen for its clear, defined criteria, offering a more precise approach compared to other commonly used classification systems (e.g., open vs. closed sports). These sport groups include team, speed/strength and precision/skill-dependent sports (McKay et al., 2022). Team sports are dynamic and strategic, requiring athletes to continuously process unpredictable information from teammates, opponents, and game-specific elements (e.g., a hockey puck or soccer ball). Speed and strength sports emphasize the ability to generate maximal force within short time frames, prioritizing power and velocity over precision during high-intensity movements (e.g., sprinting, cycling, swimming). Precision or skill-dependent sports, on the other hand, involve the execution of meticulously planned routines that demand technical accuracy, artistic expression, and/or fine motor control. Athletes in these disciplines must exhibit exceptional flexibility, balance, strength, and/or coordination to achieve precise and consistent performance. Using this classification system, one study reported that athletes from speed/strength sports demonstrated superior performance on tasks measuring cognitive flexibility, relative to team and precision/skill-dependent sports (Vona et al., 2024).

While employing sport classification systems capture broad differences in tactical, physiological, and skill-based demands across sports, it is important to note that sports within the same category may still differ in specific perceptual, decision-making, and motor demands. For example, within team sports, hockey requires rapid decision-making under physical pressure and continuous adaptation to unpredictable play, whereas volleyball involves more structured positional responsibilities and brief, constrained decision windows. As such, cognitive profiles associated with a given sport classification may reflect the shared demands of the broader category but should not be assumed to generalize uniformly to every sport within it. Given the lack of research examining broad domain-general cognitive functioning (i.e., outside of singular domains like EF) in athletes, the current study takes a multidimensional approach by examining performance on domain-general cognitive tasks among athletes from different sport classifications. Building on existing research regarding open-skill and strategic sports, we hypothesized that team sport classification would be associated with superior cognitive performance across the domains measured compared to those in speed/strength and precision/skill-dependent sports across the assessed cognitive measures. The second objective was to examine the distribution and frequency of high scores across sport classifications. As previous research has suggested, athletes involved in strategic and open-skill sports must sustain both physical and cognitive functioning over extended periods, often under high-pressure and fatigue-inducing conditions, all while making effective decisions [e.g., (Koch and Krenn, 2021; Voss et al., 2010)]. For this reason, it was hypothesized that team sport athletes would consistently achieve the greatest proportion of high scores.

## Methods

### Participants

A total of 188 high-performance athletes from a regional sport training and development organization (*n*_Female_ = 109, *n*_Male_ = 79; Mean age ± SD: 16.56 ± 1.95 years) took part in the current study. Participants were recruited between 01/12/2022 and 03/12/2024. A high-performance athlete was defined as an individual affiliated with a regional sport training and development organization, competing at the provincial or national level, and/or those on a developmental pathway to international-level competition. It is important to note that normative data based on age and sex were available for athletes aged 13–25. As a result, 20 individuals were excluded from the sample for falling outside this age range. Participants included in the final sample had not experienced a concussion in the past 6 months and were not actively recovering from one at the time of testing. Only those who completed the full cognitive battery were included in the analysis. No *a priori* sample-size or power calculation was conducted. Given the difficulty of recruiting high-performance athletes from developmental sport programs, the current sample reflects a convenience sample of all eligible athletes who completed cognitive testing within the study period, rather than a sample size determined by statistical power considerations.

### Sport classification

This study included high-performance athletes from 19 different sports that were organized into three distinct classifications based on employing the sport classification system proposed by McKay et al. (2022). The three classifications included in the current study are team (*n*_Female_ = 61, *n*_Male_ = 33; Mean age ± SD: 16.59 ± 1.10 years), speed/strength (*n*_Female_ = 17, *n*_Male_ = 21; Mean age ± SD: 18.37 ± 2.11 years), and precision/skill-dependent sports (*n*_Female_ = 31, *n*_Male_ = 25; Mean age ± SD: 15.30 ± 2.04 years). Athletes in these sports must demonstrate exceptional flexibility, balance, strength, and power to execute precise movements. For a full list of sports and the respective athlete information (number of participants, mean age, etc.) please see Table 1.

**Table 1:** Sample characteristics.

Sport category	Sport	Sample size			Age
		All	Female	Male	Mean ± SD
Precision	Diving	9	6	3	15.11 ± 1.69
	Figure Skating	3	2	1	17.33 ± 4.93
	Sailing	4	2	2	18.75 ± 2.36
	Ski Moguls	2	1	1	14.5 ± 2.12
	Artistic Swimming	20	20	0	14.35 ± 1.49
	Freestyle Ski	18	0	18	15.44 ± 0.85
	Total	56			
Speed/strength	Athletics	9	6	3	19.33 ± 1.00
	Canoe/Kayak	3	1	2	18.00 ± 1.00
	Cross Country Skiing	4	1	3	20.50 ± 2.64
	Cycling	17	6	11	17.5 ± 2.31
	Para Athletics	1	1	0	18.0 ± NA
	Swimming	3	2`	1	17.00 ± 1.00
	Rowing	1	0	1	20.00 ± NA
	Total	38			
Team	Basketball	8	7	1	15.36 ± 1.17
	Beach Volleyball	9	7	2	16.77 ± 0.83
	Curling	8	5	3	18.75 ± 1.03
	Hockey	21	21	1	16.50 ± 0.51
	Rugby	23	12	11	15.87 ± 1.01
	Indoor Volleyball	25	10	15	16.40 ± 0.77
	Total	94			

### Materials

#### Cognitive tests

Athletes overall cognitive functioning was assessed using twelve tasks from the Creyos cognitive battery (www.creyos.com; previously Cambridge Brain Sciences), a validated neuropsychological battery used to assess domain-general cognition. The cognitive battery evaluated 12 cognitive processes. Across each task, higher scores were indicative of greater performance. A comprehensive description of each test and the cognitive processes measured can be found in Appendix A. This battery has been used to assess cognition in a variety of individuals from different age groups (e.g., 18–80) and intellectual abilities [e.g., healthy controls vs. mild cognitive impairment; (Owen et al., 2010), as well as in athletes from various ages and classifications of sport-expertise (Brewer-Deluce et al., 2017; De Waelle et al., 2021; Kamali et al., 2023)]. The Creyos cognitive battery has demonstrated strong test–retest reliability and convergent validity in numerous large-scale studies (Hampshire et al., 2012).

### Procedure

Before completing the cognitive assessment, all participants provided written informed consent. Prior to initial testing, participants were asked to use a standard laptop accompanied with a standard mouse, either at the training facility or the participants’ homes. Each cognitive task was preceded by standardized written and pictorial instructions, which participants reviewed before completing a practice trial to familiarize themselves with the task demands. Standardized pictorial and written instructions preceded each cognitive test. Every participant had to complete at least one mandatory practice trial before each test to ensure procedural consistency across settings. Participants completing the battery at home were asked to complete the cognitive battery in a private room free that is free from distractions, during the daytime, with all tests completed in one succession without interruption, mirroring the administration used at the regional testing facility. Tests were presented in a randomized sequence to counterbalance test order, and the battery took approximately 40 min to complete. Participants were not required to modify their usual routines (e.g., caffeine consumption, or sleep) prior to testing, however they were asked to not engage in strenuous exercise prior to testing. The study and experimental protocol were conducted and approved by the Human Participants Review Sub-Committee of York University’s Ethics Review Board. All methods were performed in accordance with the relevant ethical guidelines and regulations. Participants 16 years and older provided written informed consent before completing the cognitive tests. If a participant was below the age of 16, a legal guardian provided consent for the athlete, and the participant provided assent.

### Statistical analyses

All analysis was carried out using the R software (R Core Team, 2025). Descriptive analyses were conducted for each cognitive test, including mean and standard deviation for continuous variables where relevant. To examine whether belonging to a particular sport group (i.e., team, speed/strength, or precision/skill-dependent) was associated with greater cognitive performance, multiple-linear regression analysis was conducted using athletes raw scores. All models met assumptions of linearity, normality, and homoscedasticity of errors. No outliers were found in the dataset. Regression analysis was conducted to enable group comparisons while also providing effect sizes, directional relationships, and coefficients that quantify how the outcome changes relative to a reference group. Team sport was chosen as the reference group because of the *a priori* hypothesis that team-based athletes would outperform athletes from other sports. No evidence of multicollinearity was observed among the predictors. To facilitate comparisons across the 12 cognitive tasks, raw scores were then converted into T-scores. These standardized scores were adjusted for age and sex using a normative dataset of approximately 5,000 non-athletes, ensuring a meaningful baseline for interpreting athlete performance across cognitive tests. To better understand the distribution of high scores achieved, multivariate base rates (MVBs) were calculated using the following percentile cut-offs: ≥75th, ≥84th, and ≥91st. These cutoffs were adopted from Karr et al. (2016), as such all cut-offs were established *a priori*. When interpreting the observed frequencies of athletes obtaining multiple high scores, we compared these frequencies with those expected under a theoretical normal distribution. For example, in a normally distributed population, approximately 25% of individuals would be expected to obtain scores in the High Average range or above (≥75th percentile) on any given measure. As such, when discussing multivariate base rates, a high score was defined by an athlete’s ability to reach or exceed a predetermined percentile cutoff (i.e., ≥75th, ≥84th, and ≥91st).

Chi-square analyses were conducted to determine if there are significant differences in the distribution of high scores across the three sport classifications, allowing for an inferential examination of whether the proportions of athletes achieving high scores varies by sport type. Some comparisons between high score thresholds could not be conducted due to an insufficient number of observations meeting the assumptions of the chi-square test (i.e., expected cell counts). As more athletes obtained high scores at or above the 75th percentile, we were able to run a greater number of comparisons between high-score thresholds. In contrast, fewer individuals reached the 91st percentile, which limited the number of comparisons that could be performed at that level. Each threshold reflects the number of high scores achieved across the entire cognitive test (e.g., one or more, two or more, etc.). Six thresholds (i.e., one or more, two or more, three or more, four or more, five or more, six or more high scores) were included in the model assessing high scores at or above the 75th percentile, four thresholds (i.e., one or more, two or more, three or more, four or more high scores) in the model for high scores at or above the 84th percentile, and three thresholds (i.e., one or more, two or more, three or more high scores) in the model for high scores at or above the 91st percentile. An alpha value of *p* < 0.05 was employed to determine level of significance.

## Results

### Relationships between sport classification and athletes cognitive functioning

Demographic information and sport information for participants is presented in Tables 1, 2. Multiple-linear regression models were conducted across twelve cognitive *tests* using athletes’ raw scores to examine the association between sport classification on cognitive functioning among high-performance athletes. To account for multiple comparisons, *p*-values were adjusted using the False Discovery Rate correction to control the risk of false positives. Specifically, when assessing the Spatial Span task (i.e., visual short-term [working] memory), sport classification explained a significant proportion of the variance in the model (R^2^ = 0.04, *F*(2, 185) = 4.63, *p* < 0.001). The precision/skill-dependent sport group (*β* = −0.57, *t*(185) = −2.69, *p* < 0.001) and the speed/strength sport group (*β* = −0.53, *t*(185) = −2.29, *p* < 0.029) had significantly lower scores compared to individuals in team sports. Similarly, when assessing the Double Trouble task (i.e., response inhibition), sport classification explained a small but significant portion of the variance (R^2^ = 0.03, *F*(2, 185) = 3.36, *p* < 0.003). The precision/skill-dependent sport group (*β* = −7.01, *t*(185) = −2.59, *p* < 0.001) had significantly lower scores compared to individuals in team sports. When analyzing the Monkey (Number) Ladder task (i.e., visuospatial working memory), sport classification showed a significant effect as well (R^2^ = 0.04, *F*(2, 185) = 2.70, *p* < 0.001). The precision/skill-dependent sport group (*β* = −1.02, *t*(185) = −2.81, *p* < 0.001) and the speed/strength sport group had significantly lower scores compared to individuals in team sports (*β* = −0.85, *t*(185) = −2.29, *p* < 0.039). Finally, the Token Search task (i.e., working memory), approached significance (R^2^ = 0.03, *F*(2, 185) = 2.84, *p* = 0.006). The precision/skill-dependent sport group (*β* = −0.77, *t*(185) = −2.31, *p* < 0.002) had significantly lower scores compared to individuals in team sports. For a more comprehensive summary of the results, please refer to Table 2.

**Table 2:** Descriptive statistics for Creyos cognitive test scores across sport groups.

Sport classification	N	Paired associates	Rotation	Odd one out	Feature match	Spatial span	Double trouble	Polygons	Spatial planning	Number ladder	Token search	Grammatical reasoning	Digit span
Precision sports													
Grand mean	56	3.52 (0.53)	92.16 (42.18)	9.29 (4.27)	114.86 (32.15)	5.68 (0.92)	21.84 (15.91)	35.64 (20.05)	21.00 (8.69)	7.57 (1.33)	7.80 (1.58)	15.75 (5.29)	6.25 (1.19)
Diving	9	3.54 (0.67)	74.56 (52.43)	10.56 (2.60)	117.89 (30.34)	5.22 (1.09)	27.78 (18.30)	34.33 (15.56)	18.44 (8.11)	7.78 (0.83)	7.56 (1.88)	14.33 (7.40)	5.89 (1.36)
Figure Skating	3	3.73 (0.12)	91.67 (20.26)	7.00 (6.93)	105.00 (34.07)	6.00 (1.00)	32.00 (19.52)	55.00 (26.46)	13.33 (5.86)	7.67 (0.58)	8.33 (2.08)	17.00 (4.36)	6.33 (1.15)
Freestyle Ski	18	3.48 (0.59)	92.67 (46.83)	7.72 (3.97)	110.78 (37.82)	5.56 (0.92)	17.28 (14.72)	33.33 (24.23)	22.28 (8.17)	7.17 (1.20)	7.67 (1.75)	15.89 (5.87)	5.78 (0.94)
Sailing	4	3.73 (0.30)	105.25 (14.77)	11.75 (4.27)	139.25 (31.72)	6.25 (0.96)	30.75 (5.32)	33.50 (15.93)	31.25 (6.40)	9.75 (2.50)	9.75 (1.50)	18.00 (4.69)	7.00 (1.15)
Ski Moguls	2	3.70 (0.14)	69.00 (14.14)	6.50 (2.12)	132.00 (16.97)	5.50 (0.71)	18.50 (26.16)	35.50 (10.61)	19.00 (2.83)	7.00 (0.00)	7.00 (0.00)	17.00 (4.24)	8.00 (0.00)
Synchronized swimming	20	3.44 (0.51)	99.40 (40.59)	10.25 (4.55)	112.05 (28.80)	5.85 (0.81)	20.30 (15.63)	35.85 (18.73)	20.30 (9.15)	7.45 (1.15)	7.65 (1.09)	15.50 (4.26)	6.50 (1.19)
Speed strength sports													
Grand Mean	38	3.75 (0.90)	97.18 (54.10)	8.21 (4.45)	121.08 (31.83)	5.71 (1.04)	26.63 (17.96)	44.18 (25.32)	22.39 (8.42)	7.74 (1.78)	8.34 (1.92)	18.26 (5.96)	6.08 (1.50)
Athletics	9	4.37 (1.32)	69.44 (35.50)	11.89 (2.26)	135.89 (33.91)	5.89 (1.17)	28.78 (20.70)	58.44 (20.50)	22.67 (7.73)	7.78 (0.67)	10.22 (2.28)	16.33 (4.53)	6.56 (1.24)
Canoe Kayak	3	3.67 (0.23)	109.00 (32.08)	3.00 (5.57)	108.00 (27.00)	5.67 (0.58)	30.00 (13.00)	21.67 (2.89)	27.67 (9.87)	7.67 (2.08)	8.33 (1.53)	16.67 (6.43)	5.33 (0.58)
Cross Country Skiing	4	4.17 (1.03)	77.25 (60.44)	9.50 (4.80)	118.75 (30.31)	5.50 (1.00)	27.75 (11.67)	42.50 (26.51)	18.25 (4.11)	9.25 (4.57)	8.00 (0.82)	20.00 (4.69)	5.25 (3.59)
Cycling	17	3.46 (0.55)	113.65 (61.36)	6.65 (3.48)	118.82 (27.87)	5.53 (1.01)	25.59 (19.33)	39.24 (26.77)	20.88 (7.92)	7.47 (1.07)	7.88 (1.11)	19.12 (6.46)	6.12 (1.17)
Para Athletics	1	2.30 (−)	37.00 (−)	8.00 (−)	91.00 (−)	5.00 (−)	3.00 (−)	19.00 (−)	10.00 (−)	7.00 (−)	4.00 (−)	10.00 (−)	5.00 (−)
Rowing	1	3.60 (−)	184.00 (−)	15.00 (−)	189.00 (−)	7.00 (−)	51.00 (−)	72.00 (−)	39.00 (−)	10.00 (−)	9.00 (−)	31.00 (−)	6.00 (−)
Swimming	3	3.63 (0.15)	93.00 (9.54)	7.33 (5.86)	93.00 (24.27)	6.33 (1.53)	21.00 (14.80)	53.33 (25.74)	29.00 (8.54)	6.67 (1.53)	7.00 (2.00)	17.00 (4.00)	6.67 (0.58)
Team sports													
Grand Mean	94	3.73 (0.61)	91.62 (42.86)	9.77 (4.11)	128.09 (33.47)	6.24 (1.47)	28.85 (15.27)	41.46 (21.44)	22.10 (8.97)	8.60 (2.63)	8.57 (2.09)	17.30 (5.72)	7.13 (3.82)
Basketball	8	3.71 (0.66)	102.00 (60.07)	7.25 (3.88)	114.00 (36.29)	6.38 (1.41)	25.25 (17.29)	36.75 (18.27)	18.25 (11.35)	8.00 (3.25)	9.00 (2.51)	18.50 (5.07)	9.38 (4.78)
Beach volleyball	9	3.53 (0.55)	98.56 (21.67)	9.33 (3.24)	133.00 (29.83)	5.78 (0.83)	36.22 (8.54)	49.67 (18.25)	20.67 (8.79)	9.33 (5.22)	7.56 (0.88)	17.22 (4.32)	6.56 (2.65)
Curling	8	3.66 (0.68)	99.75 (35.39)	7.88 (6.71)	125.88 (38.90)	5.62 (0.52)	28.50 (19.96)	41.38 (29.78)	23.88 (9.70)	8.12 (0.83)	7.50 (2.00)	18.75 (4.59)	6.25 (2.87)
Hockey - women	21	3.88 (0.67)	82.71 (42.50)	10.57 (2.79)	128.67 (35.13)	6.43 (2.40)	29.62 (14.04)	42.76 (20.98)	21.81 (8.91)	9.29 (2.78)	8.71 (1.71)	17.00 (7.25)	8.86 (6.44)
Rugby	23	3.49 (0.46)	87.43 (52.81)	9.91 (4.25)	122.65 (32.74)	6.04 (0.82)	27.91 (15.60)	40.91 (19.71)	22.74 (9.01)	8.04 (1.58)	8.43 (2.63)	16.74 (6.08)	5.78 (1.13)
Volleyball	25	3.91 (0.62)	94.52 (36.24)	10.52 (4.10)	136.04 (32.20)	6.60 (1.29)	27.68 (16.15)	39.44 (23.52)	22.92 (8.53)	8.60 (2.16)	9.16 (1.93)	17.24 (5.24)	6.68 (1.73)

Values are presented as M (SD) where standard deviations were available. Grand Mean is presented first for each sport category.

### Performance differences among sport classifications in the proportion of high scores achieved

The proportions of high scores on the Creyos cognitive battery, classified by the ≥75th, ≥84th, and ≥91st percentiles, are displayed in Table 3. These proportions were classified in two ways: (1) proportion of athletes who achieved a high score at a specific percentile rank and (2) the proportion of athletes who attained multiple high scores across the percentile rank. Across all percentile ranks (75th, 84th, 91st), athletes from every sport classification consistently achieved at least one high score. However, as the percentile rank increased, the proportion of athletes achieving high scores decreased across all classifications of sport (see Table 3). For instance, at the 75th percentile, between 83.9–94.7% of athletes obtained at least one high score, relative to only 42.9–60.5% of athletes at the 91st percentile. When examining the proportion of high scores achieved by sport type (i.e., the range of athletes achieving at least one high score from the 75th to the 91st percentile), speed/strength athletes had the highest proportion of athletes achieving at least one high score across all percentile thresholds (94.7–60.5%), followed by team (86.2–56.4%) and precision/skill-dependent athletes (83.9 −42.9%). However, as the cutoff for the number of high scores achieved increased, team sport athletes had the greatest proportion of high scores achieved across all percentile ranks. For instance, team sport athletes at or above the 75th percentile with four or more high scores (40.4%) consistently achieved a greater proportion of high scores than speed/strength (18.4%) and precision/skill-dependent athletes (23.2%). When analyzing the proportion of 4 or more high scores at the 91st percentile, athletes from team sports had a greater proportion of high scores (10.6%) relative to speed/strength (2.6%) and precision/skill-dependent sports (0%). Please see Figure 1.

**Table 3:** Cognitive differences based on sport classification.

Model	Predictor	Mean	SD	*β*	*SE*	95% CI	*t*	*p*
Model 1: spatial span	Intercept (Team)	6.24	1.47	6.24	0.13	[6.00, 6.48]	48.58	**< 0.001**
	Precision	5.67	0.91	−0.57	0.21	[−0.99,−0.15]	−2.69	**< 0.001**
	Speed/Strength	5.71	1.03	−0.53	0.24	[−1.00, −0.07]	−2.23	**< 0.002**
Model 2: number (Monkey) ladder	Intercept (Team)	8.59	2.63	8.59	0.22	[8.15, 9.03]	38.659	**< 0.001**
	Precision	7.57	1.33	−1.02	0.36	[−1.74, −0.30]	−2.81	**< 0.005**
	Speed/Strength	7.74	1.78	−0.85	0.41	[−1.67, −0.04]	−2.07	**< 0.039**
Model 3: double trouble	Intercept (Team)	28.85	15.26	28.90	1.6	[25.58, 32.11]	17.44	**< 0.001**
	Precision	21.80	15.90	−7.01	2.7	[−12.35, −1.67]	−2.59	**< 0.010**
	Speed/Strength	26.60	18.00	−2.21	3.08	[−8.29, 3.86]	−0.72	0.472
Model 4: Token Search	Intercept (Team)	8.57	2.09	8.57	0.19	[8.18, 8.96]	43.50	**< 0.001**
	Precision	7.80	1.58	−0.77	0.32	[−1.40, −0.13]	−2.28	**< 0.018**
	Speed/Strength	8.34	1.92	−0.23	0.36	[−0.95, 0.49]	−0.63	0.529

*p*-values were adjusted using the False Discovery Rate correction to control the risk of false positives.

**Figure 1:**
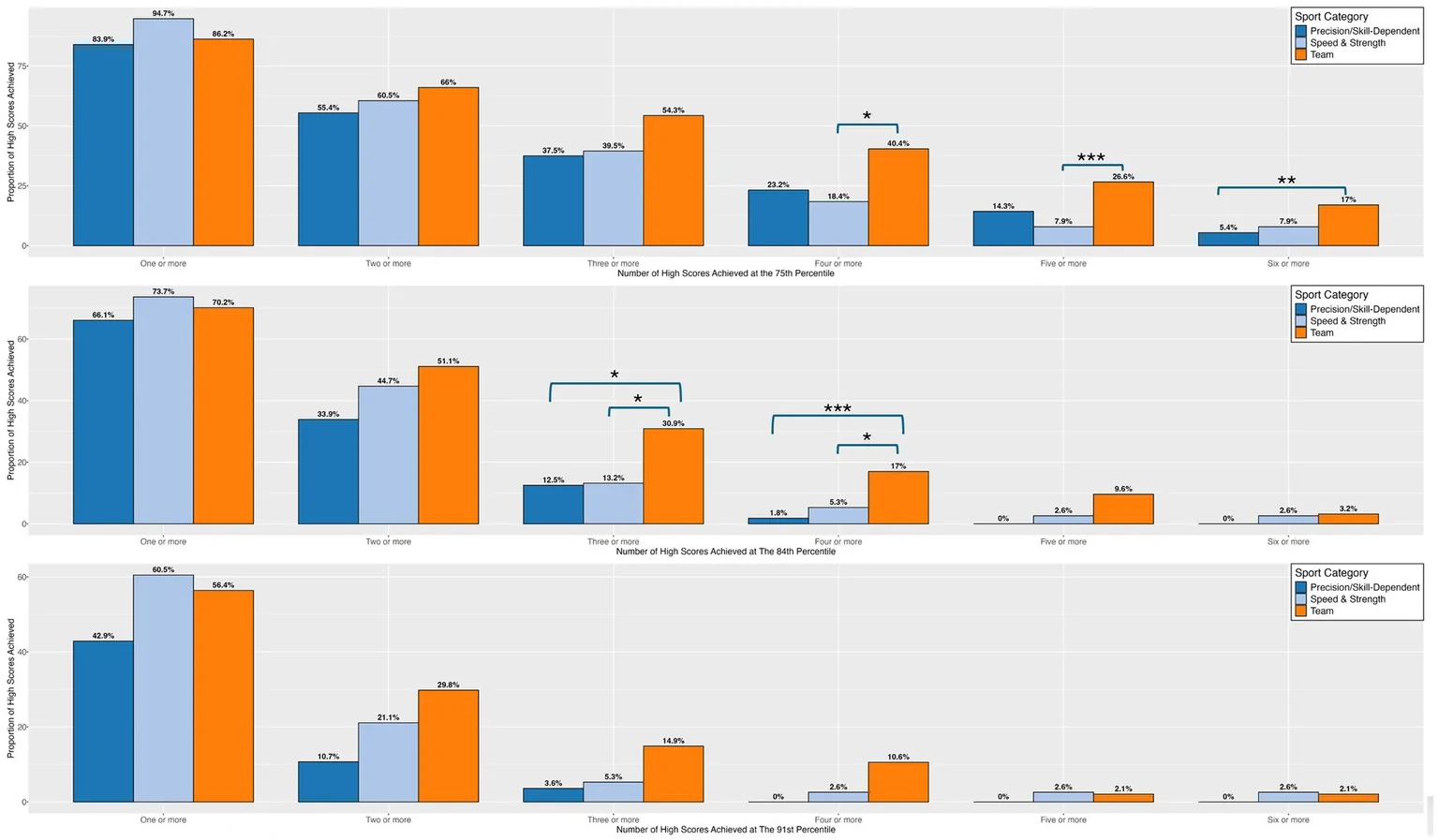
Proportion of athletes achieving one or more high cognitive scores across sport classifications at the 75th, 84th, and 91st percentile thresholds. Brackets indicate significant differences between sport categories: *p < 0.05, **p < 0.01, and ***p < 0.001.

A series of chi-square tests of independence were performed to examine the number of high scores achieved between different sport classifications. First, when comparing the number of high scores achieved across the three sport classifications at or above the 75th percentile, there was a significant difference between the expected and observed number of high scores achieved, *χ2*(10, N = 188) = 20.60, *p* < 0.002, *V* = 0.11. Post-hoc Bonferroni-adjusted comparisons revealed statistically significant differences in the number of high scores achieved, with team sports outperforming speed/strength sports at the four or more (*p* < 0.012), and five or more (*p* < 0.001) high scores cutoff, as well as outperforming precision/skill-dependent sports at the six or more (*p* < 0.004) high scores cutoff. A similar pattern emerged at the 84th percentile, *χ2*(6, N = 188) = 19.16, *p* < 0.001, *V* = 0.15. Post-hoc Bonferroni-adjusted comparisons revealed statistically significant differences in the number of high scores achieved, with team sports outperforming speed/strength at the three or more (*p* < 0.002) and four or more (*p* < 0.039) high score cutoff as well as outperforming precision/skill-dependent sports at the three or more (*p* < 0.015) and four or more (*p* < 0.001) high score cutoff. Also, when looking at the number of high scores achieved across the three sport classifications at or above the 91st percentile, there is no significant difference between the expected and observed number of high scores achieved, *χ2*(6, N = 188) = 8.69, *p* = 0.069, *V* = 0.13.

## Discussion

The current study explored the relationship between sport classification and multidimensional cognitive functioning in high-performance athletes. We hypothesized that athletes from team sports would demonstrate superior cognitive performance compared to those in speed/strength and precision/skill-dependent sports across the assessed cognitive measures. Our findings partially support this hypothesis, as team sport athletes demonstrated superior cognitive performance in tasks assessing visual short-term (working) memory, response inhibition, visuospatial working memory, and working memory, relative to precision/skill-dependent and speed/strength sports. There were no group differences for the remaining cognitive domains assessed. The second objective of the study was to examine the distribution and frequency of high scores on cognitive testing across sport classifications. We hypothesized that team sport athletes would achieve the greatest proportion of high scores. Consistent with our hypothesis, team sport athletes achieved a greater proportion of high scores relative to other sport classifications across the percentile cutoffs examined. While speed/strength athletes had the highest proportion of athletes that achieved at least one high score at the 75th percentile, team sport athletes had the highest proportion of high scores as the threshold (i.e., number of high scores achieved) increased. This result was confirmed by a series of chi-square analysis, which revealed significant pairwise differences at the 75^th^ and 84th percentiles. Specifically, team sport athletes had a significantly higher proportion of high scores than speed/strength and precision/skill-dependent athletes as the threshold for the number of high scores achieved increased.

When discussing the findings above, it is vital to highlight that it may be possible that athletes self-select their sport based on pre-existing cognitive strengths or preferences that then may develop based on training and competition demands. Given the cross-sectional nature of the data, the directionality of this relationship cannot be determined. As such, when discussing the findings of the current study, team sport athletes outperformed those in speed/strength and precision/skill-dependent sports, particularly in tasks assessing visual short-term (working) memory, response inhibition, visuospatial working memory, and working memory. The findings of the current study are consistent with previous research demonstrating that athletes in open-skill or externally-paced sports (e.g., team sports) tend to demonstrate superior cognitive performance, particularly in areas such as attention, EF, and decision-making, compared to those in closed-skill sports (Holfelder et al., 2020; Koch and Krenn, 2021; Krenn et al., 2018; Meng et al., 2019; Rahimi et al., 2022; Voss et al., 2010; Yu and Liu, 2020). These findings may support the notion that athletes in speed/strength and precision/skill-dependent sports are more closely associated with physical skills and less on higher-order cognitive functions (Holfelder et al., 2020; Voss et al., 2010). In contrast, the multifaceted cognitive demands of team sports, such as rapid decision-making, strategic planning, and real-time adaptability may be associated with superior domain-general cognitive performance (Bianco et al., 2017; Diamond and Ling, 2016; Frederiksen et al., 2014; Kida et al., 2005). This aligns with existing research suggesting that the dynamic and unpredictable nature of team, strategic, and open-skill sports, which demand continuous adaptation, quick decision-making, and strategic thinking, may be linked to superior EF (Alves et al., 2013; De Waelle et al., 2021; Heppe et al., 2016; Rahimi et al., 2022).

As team sports consistently achieved greater cognitive functioning across various tasks compared to athletes in speed/strength and precision/skill-dependent sports, it is vital to understand the demands behind these different types of sports. Individuals from speed/strength and precision/skill-dependent sports take part in activities that require fewer externally driven cognitive demands during competition (e.g., pre-planned routine, sprinting); differing cognitive demand profiles are one possible correlate of the group differences observed in cognitive performance in our study, though the current design cannot establish it as an explanatory mechanism. While the current study found evidence of superior cognitive functioning across different classifications of sport, previous research has reported mixed findings on the relationship between athletic participation and cognitive functioning (Heppe et al., 2016). These previous results may have been influenced by several factors, including variability in how specific cognitive domains were measured, the type of assessment used (e.g., computerized neurocognitive testing vs. traditional paper-and-pencil measures), and differences in the populations studied [e.g., high-performance athletes, para-athletes, or single- vs. multi-sport athletes; (Burris et al., 2019; Chiu et al., 2017; Di Russo et al., 2010; Krenn et al., 2018; Meng et al., 2019)].

When examining the data from a different perspective, specifically by analyzing the distribution and proportions of high scores achieved, we can identify athletes who consistently perform well across numerous tasks. Multivariate base-rates revealed that team sport athletes achieved a greater proportion of high scores relative to other sport classifications across two of the three percentile cutoffs examined (i.e., 75th, and 84th). While speed/strength athletes had the highest proportion of athletes who achieved at least one high score, team sport athletes had the highest proportion of high scores as the threshold (i.e., number of high scores achieved) increased. Prior studies using a clinical population have suggested that isolated high scores in specific cognitive domains are relatively common, even in the absence of superior performance across broader, domain-general tasks (Karr et al., 2016; Krenn et al., 2018).

Although this study lacked a comparison group, patterns of cognitive performance in Table 3 offer valuable descriptive insights. Assuming a normal distribution, approximately 25, 16, and 9% of individuals would be expected to score at or above the 75th, 84th, and 91st percentiles, respectively. Our results demonstrated that nearly 30% of athletes exceeded the 75th percentile on Grammatical Reasoning (verbal reasoning), Odd One Out (deductive reasoning), and Monkey Ladder (visuospatial working memory). Additionally, over 18% surpassed the 84th percentile and 13% the 91st on Rotations (mental rotation), while 24 and 13% exceeded the 84th and 91st percentiles, respectively, on Monkey Ladder. As expected, higher percentile thresholds were associated with fewer athletes achieving multiple high scores. This pattern is consistent with normative data from the Delis–Kaplan Executive Function System, a neuropsychological battery of executive functioning (Karr et al., 2019) in which high scores became less frequent at elevated thresholds but remained evident across diverse populations. Without a non-athlete control group, we cannot draw broad conclusions; however, if similar distributions were observed in a normative sample, it could suggest that athletes’ performance reflect task-specific expertise rather than superior global cognitive ability. Notably, team sport athletes maintained high performance across increasing percentile thresholds (75th–91st), which is consistent with the possibility that the dynamic and unpredictable nature of team sports relates to broader cognitive adaptability (Alves et al., 2013; Heppe et al., 2016). This may support previous findings on limited skill transfer (Furley and Memmert, 2011) indicating that while athletes may excel in certain cognitive domains, this success may not generalize across all areas.

An additional consideration, particularly relevant to younger athletes, is the role of physical development and biological maturation in athletic performance. Chronological age does not necessarily correspond to biological maturity, and substantial inter-individual variability in growth and maturation occurs throughout adolescence (Malina et al., 2015). Changes in stature, body composition, muscle mass, neuromuscular control, cardiorespiratory capacity, and metabolic function can contribute to developmental changes in physical performance, while the timing and tempo of these changes vary considerably across individuals (Stratton and Oliver, 2019). From a neuropsychological perspective, this consideration is particularly important because athletic performance reflects the interaction of cognitive, perceptual, and motor processes with the physiological capacity to execute the resulting response. Cognitive functions such as attention, executive control, decision-making, and processing efficiency may contribute to the selection and regulation of an appropriate response, but the ability to translate these processes into sport performance is also constrained by the athlete’s neuromuscular and cardiorespiratory capacities. The present findings should therefore be interpreted within this broader developmental context, particularly for younger athletes, rather than as evidence that cognitive characteristics operate independently of physical development.

When examining sport classification frameworks, researchers have explored a variety of approaches, but none has fully captured the unique cognitive demands specific to each sport (Heilmann et al., 2022). Commonly used frameworks oversimplify the cognitive complexity of sports, failing to account for the varying cognitive demands that may exist within each category. For instance, artistic swimming is often categorized as a closed-skill sport due to its predictable, rehearsed, and environment-controlled nature, involving pre-planned routines performed with minimal external variability; however, its fluid and unpredictable elements, such as synchronizing movements with changing music and maintaining precision under varying conditions call into question the appropriateness of this classification. Also sports such as tennis are labelled as interceptive, yet athletes who play tennis require strategic thinking, real time decision-making, and adaptive responses to opponents’ moves. To address this, the current study used a more refined classification framework based on the tactical, physiological, and skill demands of each sport (Table 4).

**Table 4:** Multivariate base rates of high scores achieved.

Number of high scores	75th percentile			84th percentile			91st percentile		
	PS	SS	T	PS	SS	T	PS	SS	T
No high scores	16.1	5.3	13.8	33.9	26.3	29.8	57.1	39.5	43.6
1 or more	83.9	94.7	86.2	66.1	73.7	70.2	42.9	60.5	56.4
2 or more	55.4	60.5	66.0	33.9	44.7	51.1	10.7	21.1	29.8
3 or more	37.5	39.5	54.3	12.5	13.2	30.9	3.6	5.3	14.9
4 or more	23.2	18.4	40.4	1.8	5.3	17.0	–	2.6	10.6
5 or more	14.3	7.9	26.6	–	2.6	9.6	–	2.6	2.1
6 or more	5.4	7.9	17	–	2.6	3.2	–	2.6	2.1
7 or more	1.8	5.3	8.5	–	2.6	3.2	–	2.6	1.1
8 or more	–	5.3	5.3	–	–	1.1	–	–	–
9 or more	–	2.6	1.1	–	–	–	–	–	–
10 or more	–	–	1.1	–	–	–	–	–	–
11 or more	–	–	1.1	–	–	–	–	–	–
12 high scores	–	–	–	–	–	–	–	–	–

Sport classifications are presented in the following order: Precision/skill dependent (PS)/speed-strength (SS) /team (T). All values represent cumulative percentages, except for the ‘No high scores’ row, which indicates the percentage of participants who did not exceed any high score cutoffs. The base rate calculation includes only participants with complete data.

### Limitations

This study has several limitations that must be acknowledged, the first being that this study employed a cross-sectional design in which athletes were not assessed prior to entering their respective sports, nor were they randomly assigned to sport classifications. As such, the observed associations between sport classification and cognitive functioning cannot be interpreted as evidence that sport participation produced or transferred to these cognitive differences. Superior performance among team sport athletes may instead reflect self-selection into sport based on pre-existing cognitive strengths, or other individual characteristics unrelated to sport participation. Secondly, group-specific data such as level of education, training history, years of sport participation, weekly training volume, concussion history, sleep, and medication use were not collected. While age and sex were accounted for using age and sex-adjusted normative scores, and all athletes were drawn from a comparable competition tier within the regional training program, unmeasured factors such as training volume or concussion history may still influence cognitive performance and should be considered in future research. Also, the distribution of athletes in each sporting group was not evenly distributed which may limit the generalization of these findings. As it is preferable to take an individualized approach to understanding athletes’ cognition to accurately capture sport-specific cognitive demands, having sufficiently powered sample sizes of high-performance athletes in a singular sport is extremely challenging; as such, the current study had to employ a convivence sample of all eligible athletes within the testing period (Vona et al., 2024). Consequently, it remains unclear whether the results of this study were driven by a particular sport. Due to sample size constraints within individual sports, we were unable to conduct sensitivity analyses to determine whether results were disproportionately driven by any single sport within a classification; this remains an important direction for future research with larger, sport-specific samples. Although this classification is improved over the static/interceptive/strategic framework, some pitfalls are still present. For instance, team sports such as hockey and volleyball have very different requirements of their athletes based on both their own actions and those of teammates and opponents in real-time, yet these domains are grouped together as a team sport despite the varying cognitive demands. For instance, hockey requires rapid decision-making under physical pressure, and dynamic adaptation to unpredictable play, whereas volleyball emphasizes anticipatory processing, structured positional responsibilities, and brief, highly constrained decision windows. To overcome such pitfalls, a more individualized approach to understanding sport-specific effects is necessary. This can be achieved by studying a group of athletes from a single sport, which allows researchers to examine within-sport differences in cognitive performance that may vary based on positional roles or physiological characteristics. However, we recognize the challenges involved in such research, including access to specific sports of sufficient sample sizes. While the current study adopted a group-level approach to examining athletes’ cognition, future research is encouraged to take an individual-level approach, as this can offer personalized insights into how cognitive functioning supports sport-specific performance.

Also, the age range of 13–25 was determined by the availability of normative data for this population. As a result, our findings do not extend to athletes outside this age group, limiting the development of broader cognitive profiles across different stages of athletic development. Additionally, neurodevelopmental diagnoses such as attention-deficit/hyperactivity disorder (ADHD) or autism spectrum disorder (ASD) were not assessed and therefore could not be accounted for in the analyses. An additional limitation is the lack of real-time monitoring for fatigue-related disengagement, as some athletes completed the testing on their own due to isolation requirements during the COVID-19 pandemic. However, analyses conducted both including and excluding these participants revealed the same patterns of results, suggesting that this factor did not meaningfully affect the findings. As such, this may have resulted in reduced effort on later tasks, potentially affecting performance outcomes. Finally, the present study did not directly assess maturational status or physical characteristics; consequently, future research should consider integrating measures of biological maturation, physical capacity, and neuromuscular performance alongside cognitive measures to more comprehensively characterize the factors contributing to athletic performance. Future research is also encouraged to take a longitudinal approach to better understand how sport classification impacts athletes’ cognitive development and performance over time.

## Conclusion

The current study contributes to our understanding of the relationship between sport classification and multidimensional cognitive functioning in high-performance athletes by employing inferential and neuropsychological methodologies to assess cognitive performance across different sport classification. This approach emphasizes the importance of taking a holistic perspective in examining cognitive profiles, highlighting how understanding athletes’ classification of sport and the interrelationships among cognitive domains can possibly inform athlete identification, training strategies, as well as performance optimization across different classifications of sport.

## Data Availability

The raw data supporting the conclusions of this article will be made available by the authors, without undue reservation.
