# Supplementary material for: Multidimensional cognitive functioning across sport classifications: insights from high-performance athletes
Source: Front Psychol. 2026 Sep 23;17:1837191. doi: 10.3389/fpsyg.2026.1837191 (PMC13642147; doi:10.3389/fpsyg.2026.1837191)
Supplement: Supplementary file 1 [file Supplementary_file_1.docx]

**Appendix A**

**Double Trouble (DT)**

This task is an iteration on the classic Stroop test [1] that assesses participants’ ability to inhibit information. In DT, a target appears on the screen in either a red colour or blue colour. Participants are tasked to select the probe word that corresponds to the colour that the target word is drawn too. The colour mappings can either be congruent (i.e., every word correctly describes the displayed colour), incongruent (i.e., target word or the probed word is displayed in opposite colours), or double incongruent (i.e., target and probe are both displayed in colours that are opposite to what is being described). Each participant has 90 seconds to complete as many trails as they can within the specified time. A correct response is worth one point while an incorrect response removes one point from the participant's total score.

**Spatial Planning**

Spatial planning is a task that is based on the Tower of London [2] which is primarily used as a measure of spatial working memory/planning. In this task, numbered beads are placed on a tree, and participants are asked to rearrange the beads in ascending numerical order. Participants are given four minutes to solve as many puzzles as possible. For every puzzle completed, the trial increases in difficulty each time. A trial is stopped if participants make more than twice the required number of moves required to solve the problem. For each completion of a puzzle, the participants’ final score increased by two times the difference between the minimum number of moves required and the number of moves made.

**Odd One Out**

Odd One Out is a task based on reasoning problems from the Cattel Culture Fair intelligence test [3]. Nine groups of coloured shapes are presented in a grid format consisting of different colours, shapes and number of items present. These items define each group and are related to one another through a set of rules. Participants are asked to spot a pattern and identify the group that does not pertain to the current set of rules. Participants are given 90 seconds to solve as many problems as possible. Correct responses result in one point while an incorrect response reduced the total score by one point.

**Grammatical Reasoning**

Grammatical reasoning is a task that assesses verbal reasoning based on Baddeley’s three minute Grammatical Reasoning test [4]. For each trial, participants are presented with two shapes on the screen with a corresponding statement. Participants are then asked to indicate if the statement correctly describes the shapes displayed. Participants are given 90 seconds to complete as many responses as possible. Correct responses result in a total score of one point while incorrect responses result in a reduction of one point.

**Polygons**

Polygons is a task based on the interlocking pentagons test that assesses visuospatial functioning [5]. For each trial, participants are presented with overlapping wire-framed polygons on the left side of the monitor. Participants are tasked with identifying if the shape to the right is identical to one of the two overlapping ones on the left side of the screen. Correct responses increase the difficulty of the subsequent trial (i.e., differences in overlapping polygons will be less distinct). Each participant has 90 seconds to complete as many trials as possible.

**Digit Span**

Digit span is a verbal working memory task based on the revised Wechsler Adult Intelligence Scale [6]. This task presents participants with a sequence of digits displayed one after another in the centre of the monitor. Participants must repeat these digits by selecting a sequence of digits via an on-screen keyboard. Difficulty is dynamically varied as the previous tests mentioned. The test ends after three mistakes made. Individuals score is the length of the longest digit sequence recalled.

**Rotations**

Rotations is task designed to assess individuals’ mental rotation skills. Each trial consists of two groups of coloured squares (each containing n squares) beside one another. One group is rotated by a multiple of 90 degrees. Each group is either identical or differs by position, one item at a time. Participants must indicate if the groups presented match. Correct responses increase the final score by n while subsequent trial has groups of n + 1 squares. If participants record an incorrect response, the total score decreases by n, and the next trial has groups of n – 1 squares.

**Token Search**

Token Search assesses participants working memory skills during search related tasks (e.g., [7]). This task presents participants with a set of boxes with a hidden green token in one of the boxes displayed on a grid. Participants are then asked to find the token by clicking one box at a time. Once the token is found, the token is then placed in a new box. A token will not be in a box that previously held a token, thus requiring participants to remember previous correct responses. A wrong answer occurs when a participant chooses a box that was a correct answer previously. A new trial begins when participants select a box that has not been previously used to identify a token. The trial ends after three errors and participants’ maximum level are their final score.

**Paired Associates**

Paired Associates is a task designed to measure participants’ episodic memory. A set of boxes will appear on the participants’ monitor at random and are to open one after another to reveal an icon after they close. These icons are to be displayed sequentially in the centre of the screen and athletes will then be tasked to select a box that contains that item. If participants remember all the icon-location pairs the next trial will add an additional box. If an error is made, the following trial will have one less box. The task ends after three errors. Participants’ maximum number of matched pairs in their total score.

**Spatial Span**

Spatial Span is a visual short-term (working) memory task based on the Corsi block-tapping test. In the current task, sixteen purple blocks are displayed in a grid, and a selected number of boxes turn green one at a time (900ms per green square). Participants are then asked to repeat the sequence by clicking the boxes in the same order. Once more, difficulty varies dynamically. The test ends after three errors. Participants’ final score is the length of the longest successful sequence recalled.

**Feature Match**

Feature Match is a perceptual discrimination task in which two boxes appear on the screen, each displaying an array of abstract shapes. Participants must determine whether the arrays are identical or different and respond by clicking the appropriate button. Task difficulty adapts in real-time based on participant performance, maintaining an optimal level of challenge throughout.

**Monkey (Number) Ladder**

Monkey (Number) Ladder is a visuospatial working memory task in which numbered boxes briefly appear in random locations on the screen. After the numbers disappear, participants must recall the sequence by clicking the boxes in ascending numerical order. The task adapts in difficulty based on performance, with scores reflecting the average number of boxes correctly remembered.

**Bibliography**

1. Stroop, J. R. Studies of interference in serial verbal reactions. Journal of Experimental Psychology 18, 643–662 (1935).

2. Shallice, T. Specific impairments of planning. Philosophical Transactions of the Royal Society of London. B, Biological Sciences 298, 199–209 (1982).

3. Cattell, R. B. The dimensions of culture patterns by factorization of national characters. The Journal

4. Baddeley, A. D. How Does Acoustic Similarity Influence Short-Term Memory? Quarterly Journal of Experimental Psychology 20, 249–264 (1968).

of Abnormal and Social Psychology 44, 443–469 (1949).

5. Folstein, M. F., Folstein, S. E. & McHugh, P. R. “Mini-mental state”. Journal of Psychiatric Research 12, 189–198 (1975).

6. Wechsler, D. The psychometric tradition: Developing the wechsler adult intelligence scale. Contemporary Educational Psychology 6, 82–85 (1981).

7. Collins, P., Roberts, A. C., Dias, R., Everitt, B. J. & Robbins, T. W. Perseveration and Strategy in a Novel Spatial Self-Ordered Sequencing Task for Nonhuman Primates: Effects of Excitotoxic Lesions and Dopamine Depletions of the Prefrontal Cortex. Journal of Cognitive Neuroscience 10, 332–354 (1998).
